# A Novel Approach of Administering Cranberry Extract Into 3D-Printed Denture Bases for the Prevention of Denture-Induced Stomatitis: An Observational Study

**DOI:** 10.7759/cureus.79438

**Published:** 2025-02-22

**Authors:** Aishwarya Roy, Saravanan M, Vishal Reddy, Muthukumar B

**Affiliations:** 1 Prosthodontics, SRM Dental College and Hospital, Ramapuram, Chennai, IND; 2 Prosthodontics and Implantology, SRM Dental College and Hospital, Ramapuram, Chennai, IND

**Keywords:** 3d printing, adhesion, antifungal, biofilm, cranberry extract, denture bases, denture-induced stomatitis

## Abstract

Background: Complete dentures play an important role in restoring oral function and aesthetics, yet they may contribute to denture stomatitis, necessitating improved materials and hygiene practices. Cranberry extract, known for its antifungal properties, presents a promising avenue for preventing stomatitis when incorporated into novel denture materials.

Aims and objectives: This research aimed to assess the efficiency of cranberry extract-infused stereolithography (SLA) 3D-printable resins in preventing denture-induced stomatitis and compare their mechanical properties with conventional heat-cured denture base polymers.

Materials and methods: Fifteen patients aged 45 to 60 with completely edentulous maxillary and mandibular arches received two sets of dentures: control dentures made from heat-activated polymethyl methacrylate (PMMA) and treatment dentures made from cranberry-infused 3D-printed resin. Candidal colony-forming units (CFUs) and confocal microscopy were used to assess biofilm formation on 30 samples. For the evaluation of mechanical properties, 30 samples were made in each group, and the flexural strength and fracture toughness were examined for both the control and test groups.

Results: Significantly fewer CFUs were observed in 3D-printed dentures compared to PMMA dentures at 10^4^ concentrations (p=0.03). Biofilm thickness was significantly lower in 3D-printed dentures (p=0.039), but volume fraction biofilm exhibited no discernible change (p>0.05). Surface coverage was significantly reduced in 3D-printed dentures (p=0.028). Flexural strength was higher in 3D-printed samples (124.25±2.67 MPa) compared to PMMA (109.76±9.35 MPa), with a statistically significant difference. Fracture toughness was also significantly higher in 3D-printed dentures (1.60±0.12) compared to PMMA (1.38±0.95) (p=0.028).

Conclusion: Cranberry-infused 3D-printable resins demonstrate promise in dropping *Candida* adhesion and biofilm formation, potentially lowering the risk of denture stomatitis. Moreover, these resins exhibit superior mechanical properties compared to conventional denture base polymers, suggesting a potential alternative for prosthodontic applications.

## Introduction

The phenomenon of complete edentulism, denoting the total loss of all teeth, is observed worldwide. As per the criteria outlined by the World Health Organization, individuals experiencing complete edentulism are classified as disabled, handicapped, and physically impaired due to their inability to effectively chew and speak [1]. The onset of edentulism stems from various biological factors like oral cancer, trauma, periodontal diseases, and dental decay. Within the geriatric demographic aged over 65, the ratio of edentulous to dentate individuals stands at 2:1. Research by Al-Rafee revealed a decline in edentulism among teenagers and middle-aged adults in Brazil, yet a projected rise in the elderly, reaching over 64 million by 2040. This underscores the persistent and growing need for functional restoration among edentulous patients globally [2].

Edentulism is associated with several comorbidities that significantly impact individuals. A study by Felton linked edentulous patients to poor dietary habits, nutritional deficiencies, osteoporosis, hypertension, and coronary artery disease. Moreover, edentulism is correlated with smoking and smoking-related ailments, compromised health-connected QoL (Quality of Life), and increased risk of various systemic diseases, including diabetes, cardiovascular conditions, and chronic kidney disease [3]. Successful rehabilitation for edentulous patients often involves complete denture therapy, a traditional treatment option, especially for individuals facing systemic, anatomic, or financial constraints. Prolonged denture use can lead to immunodeficiency in older individuals due to systemic diseases and nutritional deficiencies, resulting in residual ridge resorption and denture-induced stomatitis [4].

Conventional complete removable dentures are typically made from polymethyl methacrylate (PMMA), which is known for its simplicity of processing, biocompatibility, repair, and aesthetic appeal [5]. However, PMMA has drawbacks, such as high shrinkage, susceptibility to microbial colonization, and degradation of mechanical properties over time. Novel materials and manufacturing techniques, including digital dentistry, are emerging to address these limitations [6].

*Candida*-associated denture stomatitis (DS), affecting approximately 60% of prosthesis wearers, is a common inflammatory condition. *Candida albicans*, a commensal organism in the oral cavity, becomes virulent under immunodeficient conditions, causing candidiasis with varied clinical manifestations [7]. The pathogenesis of *Candida*-related DS is complex, involving host-related and *Candida*-specific factors affecting adhesion and proliferation in host epithelial tissues [8]. The American cranberry (*Vaccinium macrocarpon*) is rich in (poly)phenols, which possess a range of advantageous qualities, such as antioxidant, antiviral, antibacterial, and anti-inflammatory actions. Cranberry proanthocyanidins (PACs) have demonstrated strong activity against *Candida* *albicans* biofilms, suggesting their potential in preventing urinary catheter infections [9]. A study by Girardot et al. demonstrated the promising potential of cranberry extracts and juice fractions, particularly those enriched in proanthocyanidins, in preventing dental caries by inhibiting the adhesion and biofilm formation of *Candida* species. This research suggested that incorporating cranberry into the diet could serve as an effective natural strategy for dental caries prevention [10].

Therefore, based on the aforementioned details, this research aims to assess the incorporation of cranberry (*Vaccinium macrocarpon*) extract into stereolithography (SLA) resin 3D-printed denture bases to prevent denture-induced stomatitis and to compare the mechanical characteristics, such as fracture toughness and flexural strength with those of conventional PMMA denture bases.

## Materials and methods

The procedures followed were in accordance with the ethical standards of the responsible committee on human experimentation (institutional or regional) and with the Helsinki Declaration of 1975, as revised in 2000. This study obtained ethical clearance from the SRM Dental College Institutional Review Board (SRMDC/IRB/2021/MDS/No.201). It was a crossover observational study conducted on outpatients from the Department of Prosthodontics. The study started on February 20, 2022, and was completed on March 10, 2024.

The inclusion criteria are Class I patients under the PDI (Prosthodontic Diagnostic Index) classification of complete edentulism, age group of 45-60 years, both male and female patients, and previous denture wearers (conventional heat-cured PMMA). Exclusion criteria are patients with Class II, III, and IV completely edentulous arches; patients with temporomandibular disorders (TMDs), trauma, systemic conditions (diabetes mellitus, hematological disorders, blood dyscrasias), local lesions, xerostomia, and patients who smoke tobacco.

The study included a sample size of 15 participants. A power analysis was done to calculate the sample size. The standard deviation was determined to be 4.8, the sample mean was found to be 80, and the population mean was 82.4. The alpha error was found to be 5%, with a power value of 80%. The required sample size (n) was 31. Given that it was an in vivo study involving 15 patients, we have taken the sample size (n) to be 30. The power analysis is presented in Table 1.

**Table 1:** Power analysis details

Alpha Error (%)	Power (%)	Sample Size (n)
1	70	39
	80	47
	90	60
5	70	25
	80	31
	90	42
10	70	19
	80	25
	90	34

The sample allocation employs a crossover design. Participants wore both conventional and cranberry-infused 3D-printed dentures in a sequence, with conventional dentures used first, followed by 3D-printed dentures after three months. This study employed a single-blind design, wherein the researchers were aware of group allocation while the participants remained blinded.

The primary outcome measures checked were the colony-forming units (CFUs) of *Candida albicans* in saliva samples collected after three months of denture use. The secondary outcomes checked were the flexural strength, fracture toughness of denture base materials, and biofilm formation on dentures assessed by confocal microscopy.

**Figure 1:**
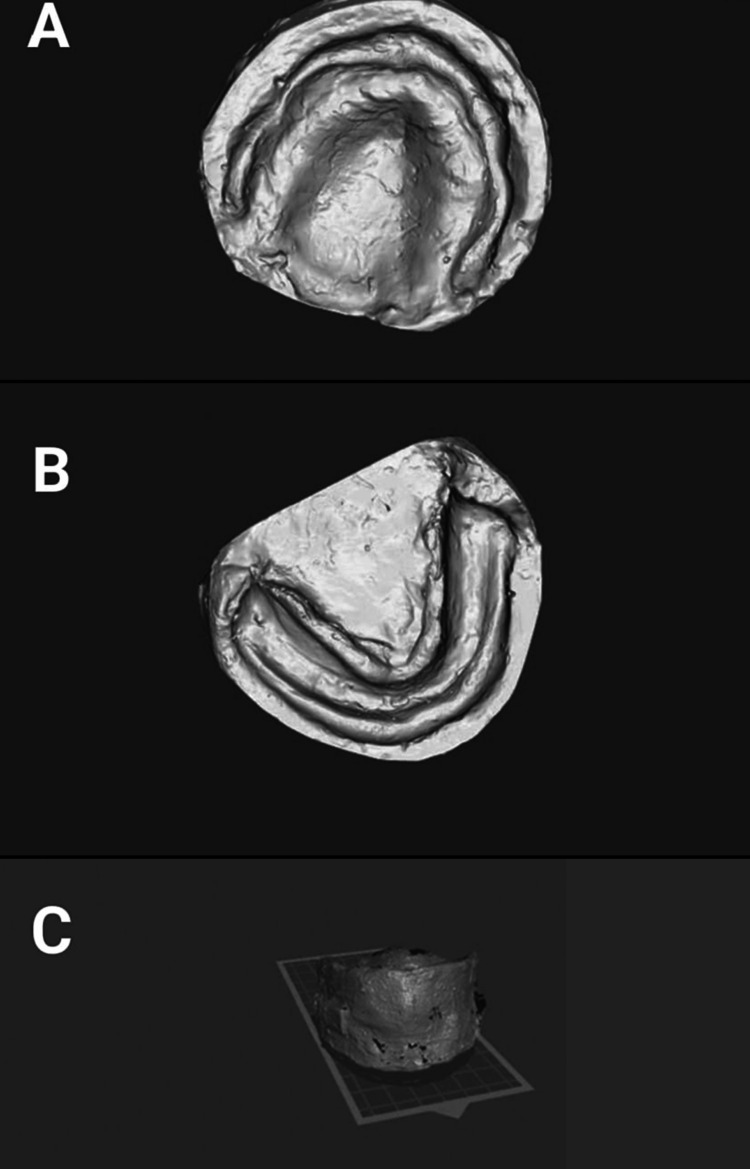
Scanning of the casts (A) Upper cast; (B) lower cast; and (C) jaw relation scan

Conventional dentures were fabricated following established techniques using heat-polymerized denture base resin. Cranberry-infused 3D-printed dentures were fabricated by creating digital scans of master casts and jaw relation designs (Figure 1) using dental design software.

**Figure 2:**
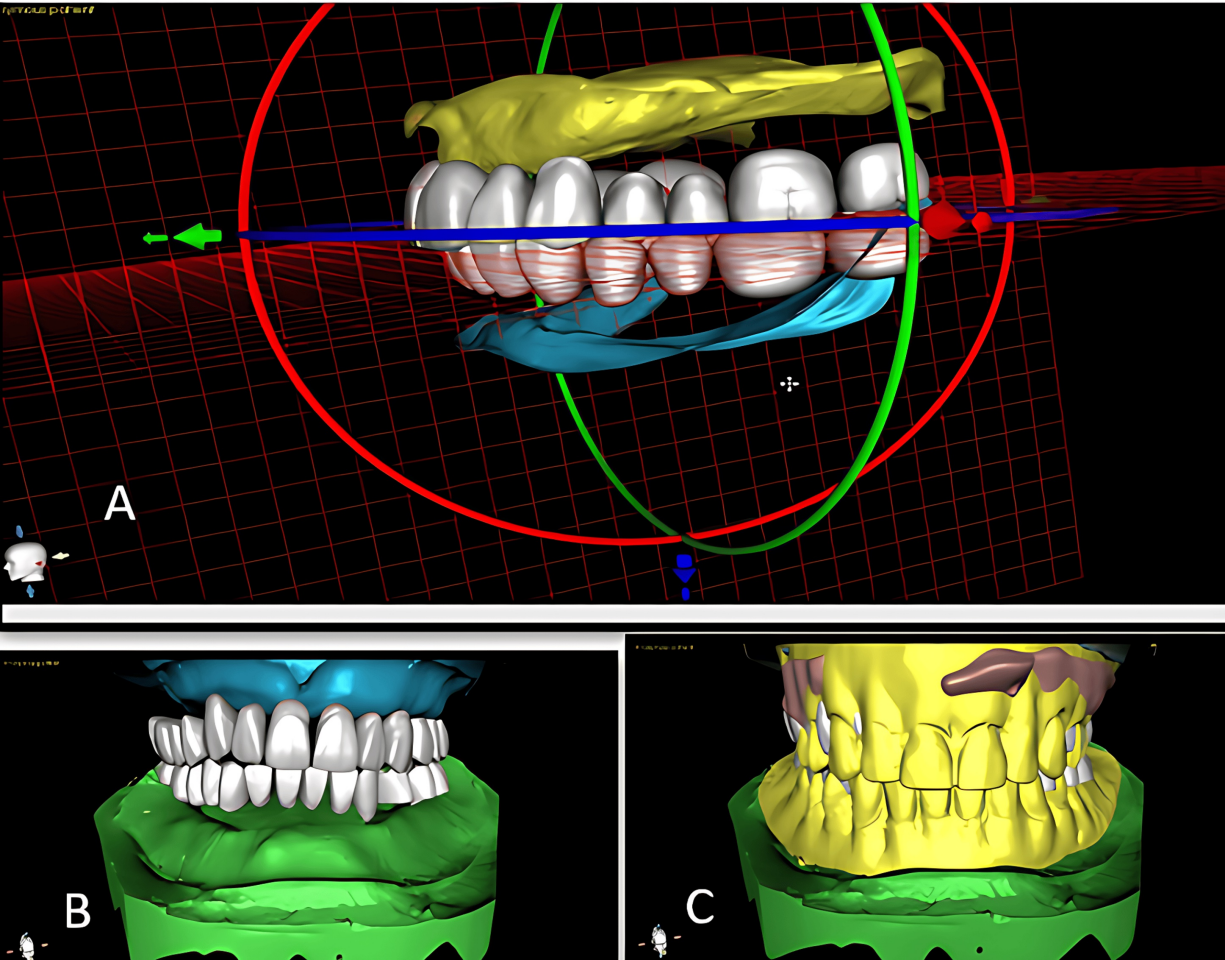
Digital teeth setting using BlueSkyBio Digital software (A) Checking of orientation jaw relation; (B) establishing the occlusion and reconfirming the position of the teeth; (C) fabrication of a digital model after marking the borders of denture extension

Digital teeth setting was done using BlueSkyBio Digital software (Blue Sky Bio, LLC, Libertyville, IL, USA) (Figure 2).

**Figure 3:**
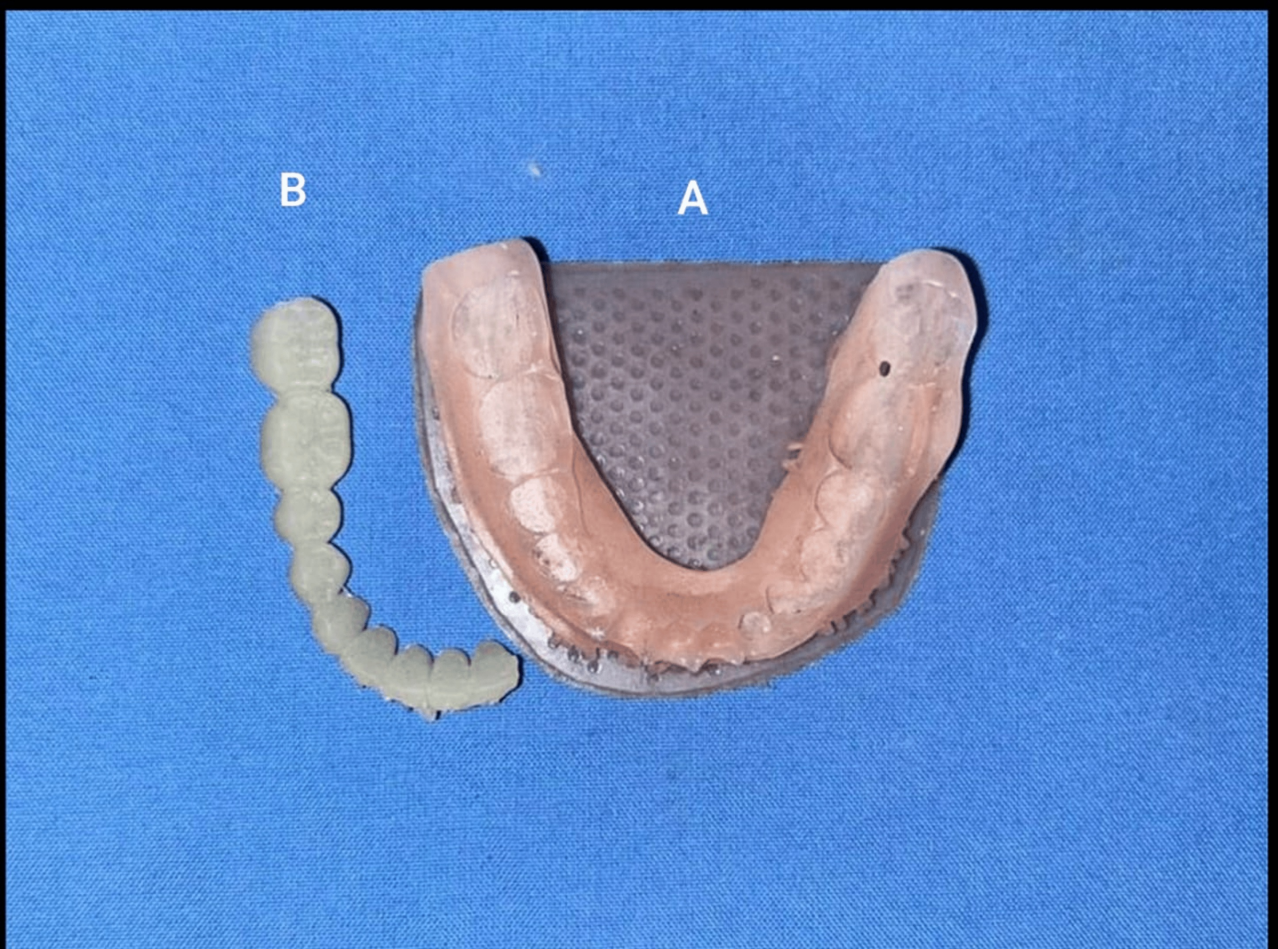
The 3D-printed parts (A) Denture base; (B) associated teeth

Printing was done with digital light processing (DLP) (Figure 3), using cranberry-infused resin, followed by post-curing.

**Figure 4:**
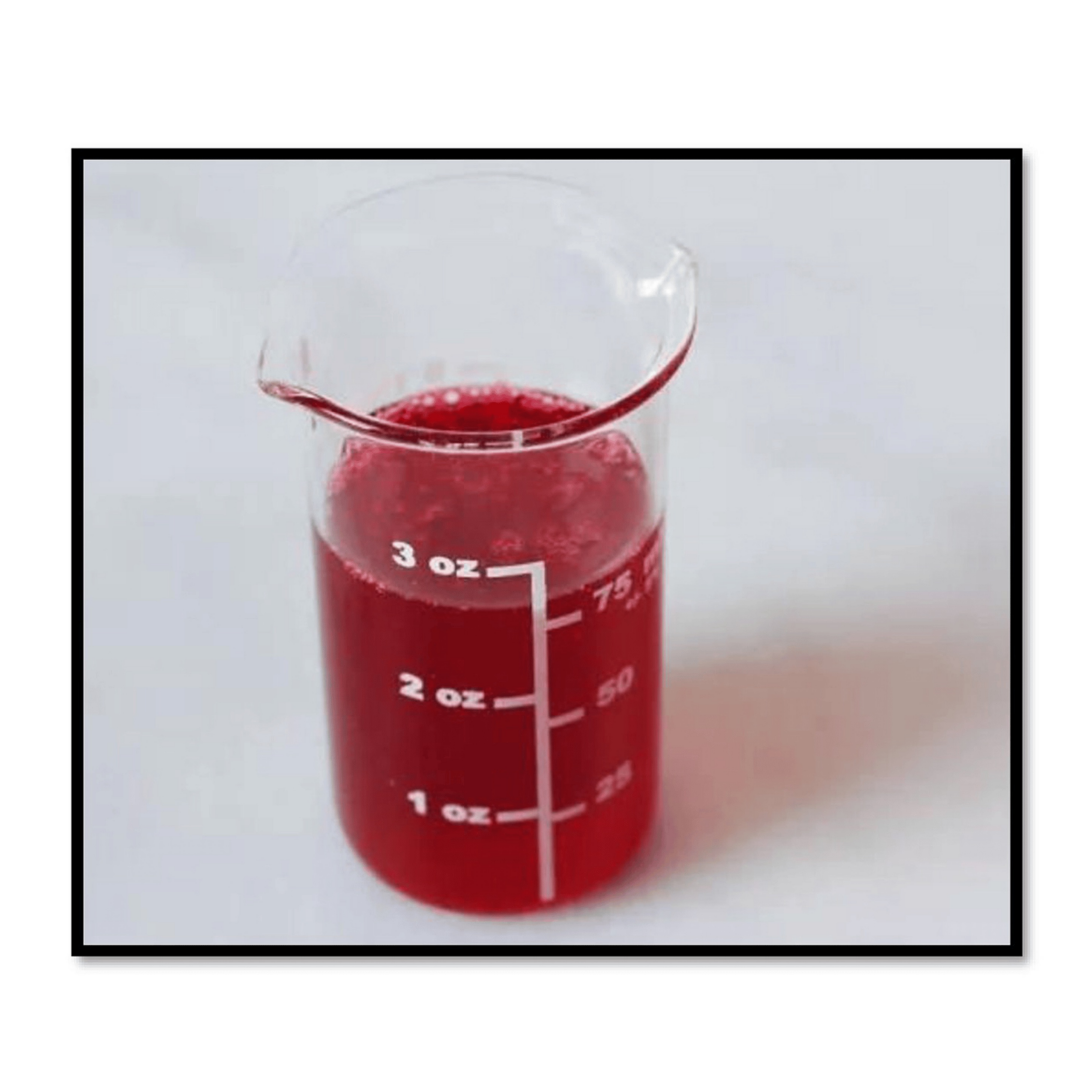
Double-filtered cranberry extract from freeze-dried cranberries Content analysis by HPLC-DAD was not conducted. The content of proanthocyanidins was standardized using a reference article [14]. HPLC-DAD: high-performance liquid chromatography with diode-array detection

The standardized method of mixing the cranberry extract into the 3D-printed denture base resin is described. A total of 200 g of freeze-dried cranberry (Figure 4) was subjected to compression to release the extract, which was then collected in a clear beaker. A total of 120 mg of cranberry extract contains 36 mg of proanthocyanidins, which are responsible for the antifungal activity [11].

Next, the volume of denture base resin was precisely measured based on the specific requirements of each patient. This measurement was determined using the Chitubox software (CBD-Tech, Shenzhen, China), which is essential for the 3D printing process. To enhance the strength and fine-tune the volume, the flexible resin was added at a concentration of 10% by weight [12].

Subsequently, this resin mixture was transferred to a larger beaker, and the cranberry extract was carefully added, drop by drop, at a ratio of 120 mg of extract per 1 mL of 3D-printable resin. The cranberry extract was infused in the 3D-printable resin before printing. The blending process was conducted using a magnetic stirrer (MX-1, Sibata Scientific Technology Ltd., Sōka-shi, Japan) operating at 1500 rpm, and continuous visual checks were performed to ensure the homogeneous mixing of the components.

**Figure 5:**
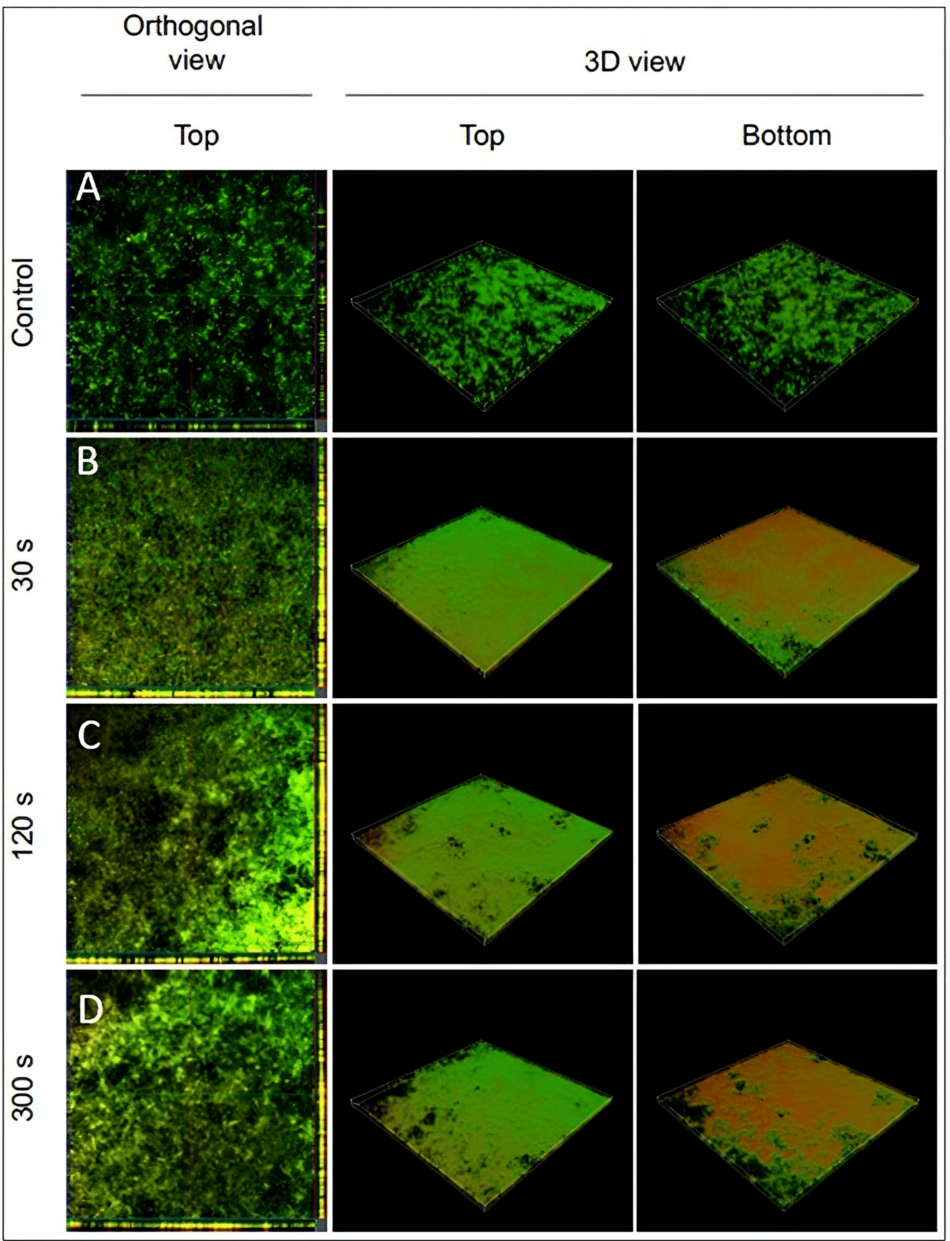
Z-stack images obtained from confocal microscopy The image shows confocal laser microscopy images of LIVE/DEAD-stained biofilms following treatment for varying durations (30, 120, and 300 seconds). The control group (A) shows a dense, predominantly green biofilm, indicating a high proportion of live bacteria. Following treatment for 30 seconds (B), there is a noticeable shift towards yellow and red, suggesting an increase in dead or damaged cells. This effect is more pronounced at 120 seconds (C), with further reduction in green and dominance of yellow/red. At 300 seconds (D), while some green remains, the biofilm is largely composed of dead or damaged cells, as shown by the extensive yellow and red staining. The 3D views provide a spatial representation of these changes, showing the distribution of live and dead cells throughout the biofilm structure. Overall, the image suggests that the treatment effectively reduces the viability of the biofilm in a time-dependent manner.

Unstimulated saliva samples were collected after three months of denture use and analyzed for CFUs of *Candida albicans* [13]. The denture surfaces were rinsed with PBS (phosphate-buffered saline) and subsequently preserved with paraformaldehyde after three months of use for confocal microscopy analysis (Figure 5). Flexural strength and fracture toughness of denture base materials were evaluated using a universal testing machine according to ISO standards (ISO 20795.1.2013).

Statistical analysis was done with IBM SPSS Statistics for Windows, Version 21 (Released 2012; IBM Corp., Armonk, New York) at 95% CI and 80% power to the study. The Kolmogorov-Smirnov and Shapiro-Wilk tests were done to check for the normal distribution of the data. Descriptive statistics were performed in terms of frequency and percentage. An unpaired t-test was applied to compare the flexural strength, fracture toughness, CFU, biofilm thickness, surface coverage, and volume fraction of biofilm between both groups. Statistical significance was calculated at p<0.05.

## Results

A comparison of CFUs between conventional dentures (control) and cranberry-infused 3D-printed dentures (treatment) in different dilutions is presented in Table 2. The findings clearly indicate the reduction in CFUs in cranberry-infused 3D-printed dentures compared to conventional PMMA dentures.

**Table 2:** Comparison of colony-forming units between conventional dentures (control) and cranberry-infused 3D-printed dentures (treatment) in different dilutions *Statistical significance at p<0.05. The findings clearly indicate the reduction in colony-forming units in 3D-printed dentures compared to conventional PMMA dentures. PMMA: polymethyl methacrylate

Variables	Groups	N	Mean	Std. Deviation	t-value	p-value
Colony-Forming Units *10^1^*	PMMA	15	15.1333	15.85139	1.919	0.065
	3D-Printed	15	6.0667	9.13757		
Colony-Forming Units *10^2^*	PMMA	15	8.9333	12.12710	1.838	0.077
	3D-Printed	15	2.8000	4.45934		
Colony-Forming Units *10^3^*	PMMA	15	4.6667	7.17801	1.825	0.079
	3D-Printed	15	1.1333	2.16685		
Colony-Forming Units *10^4^*	PMMA	15	2.6000	3.41844	2.259	0.032*
	3D-Printed	15	.4667	1.30201		

Additionally, flexural strength, fracture toughness, biofilm thickness, surface coverage, and volume fraction of biofilm of both groups are presented in Table 3. The mean biofilm thickness of PMMA dentures was 17±12.75 mm, and for 3D-printed dentures, it was 8.53±8.17 mm. A statistically significant difference was observed in the biofilm thickness between PMMA and 3D-printed dentures (p=0.039).

**Table 3:** Flexural strength, fracture toughness, biofilm thickness, surface coverage, and volume fraction of biofilm of both groups *Statistical significance at p<0.05. N=31 for the first two parameters because it was an in vitro study. This selection is based on the results of the parent article [14] and a statistician's report. N=15 for the last three parameters due to the inclusion of 15 patients. PMMA: polymethyl methacrylate

Properties	Groups	N	Mean	Std. Deviation	t-value	p-value
Flexural strength	PMMA	31	109.7613	9.35812	-7.857	<0.001*
	3D-printed	31	124.2581	2.67591		
Fracture toughness	PMMA	31	1.3855	.09591	-7.827	<0.001*
	3D-printed	31	1.6068	.12405		
Biofilm thickness	PMMA	15	17.0	12.75035	2.165	0.039*
	3D-printed	15	8.5333	8.17546		
Volume fraction of biofilm %	PMMA	15	27.1333	12.74960	0.455	0.653
	3D-printed	15	25.0667	12.12121		
Surface coverage	PMMA	15	22.0	9.56183	2.319	0.028*
	3D-printed	15	15.0667	6.52979		

The mean volume fraction of biofilm on PMMA dentures was 27.13±12.74% and 25.06±12.12% on 3D-printed dentures. No statistically significant difference was observed in the volume fraction of biofilm between PMMA and 3D-printed dentures (p>0.05).

The mean surface coverage of PMMA dentures was 22.00±9.56%, and it was 15.06±6.52% for 3D-printed dentures. A statistically significant difference was observed in the surface coverage between PMMA and 3D-printed dentures (p=0.028).

The mean flexural strength for PMMA dentures was 109.76±9.35 MPa and 124.25±2.67 MPa for 3D-printed dentures. The treatment group had a higher flexural strength than the control group, and there was a high statistically significant difference between the two groups.

The mean fracture toughness of PMMA dentures was 1.38±0.95 MPa.m1/2, and for 3D-printed dentures, it was 1.60±0.12 MPa.m1/2. A high statistically significant difference was observed in the fracture toughness between PMMA and 3D-printed dentures (p=0.028).

## Discussion

Dentures are a common solution for edentulism; however, they may lead to DS, a fungal infection. Poor denture hygiene and compromised immune systems increase the risk of developing DS [15]. This study aimed to explore alternative management strategies to improve oral health for denture wearers.

Cranberry extract shows promise as a complementary treatment for DS due to its antifungal and anti-inflammatory properties. It disrupts *Candida* adhesion, hinders fungal growth, and reduces inflammation [11]. Studies by Sugio et al. [16] and Anitha and Rajkumar [17] support its effectiveness.

3D printing revolutionizes denture fabrication by creating highly customized prosthetics that improve fit, comfort, and function. It streamlines production compared to traditional methods. Despite initial investment costs, 3D printing can be cost-effective in the long run [18,19].

A study published by Jeon et al. detailed the process of incorporating phytoncide oil into 3D-printed denture bases. This oil was microencapsulated and mixed with a 3D-printable resin to create denture bases with antifungal properties. The results showed a significant reduction in *Candida albicans* growth on the treated denture bases, highlighting the potential of integrating natural antimicrobial agents into denture materials to enhance their clinical performance and durability​ [20].

Additionally, research on the surface characteristics of milled versus 3D-printed denture bases has indicated that while milled bases generally have superior surface properties after polishing, 3D-printed bases can achieve clinically acceptable levels of smoothness and gloss, especially when subjected to advanced polishing and coating techniques​ [21].

Recent research has made significant strides in incorporating antimicrobial agents into 3D-printed denture bases. One study utilized silver-loaded mesoporous silica nanoparticles, demonstrating enhanced antimicrobial properties and mechanical strength [22]. A systematic review analyzed various organic and inorganic antimicrobial materials, assessing their efficacy and impact on denture bases [23]. Collectively, these studies highlight the potential of innovative materials to improve the functionality and infection resistance of 3D-printed dentures.

This observational study investigated incorporating cranberry extract into 3D-printed denture resin to reduce *Candida* and mitigate DS risk. The findings aligned with the study by Anitha and Rajkumar [17] but lacked comparisons with other materials. The study provides valuable data on the mechanical and biological performance of this novel resin. However, its design limits causal inferences. Future research is needed to determine optimal dosages, treatment regimens, and the long-term efficacy of cranberry extract for DS management.

The current study focused on completely edentulous patients aged 45-60 years without systemic complications and with ideal Class I ridges, excluding those with any complications and highly resorbed ridges. The utilization of 3D-printed dentures was found to effectively reduce DS, lowering the CFUs and bacterial adherence to the denture surfaces. Furthermore, when compared with PMMA dentures, the doped 3D-printed dentures exhibited superior mechanical properties, and the addition of 10% flexible resin material was responsible for superior mechanical properties.

The clinical implications of this study underscore the fabrication of stronger, more durable dentures that enhance patient comfort and minimize the risk of mucosal irritation associated with DS. This quality improvement prolongs the lifespan of the treatment, allowing patients to maintain their oral hygiene with minimal intervention and without the need for meticulous preventive measures.

However, the study does present certain limitations. Procuring the necessary resources for some practitioners might prove challenging, and clinicians must possess comprehensive knowledge of computer-assisted design to administer this treatment effectively. Additionally, the study incorporated a 10% addition of flexible biocompatible resin, which enhanced mechanical properties and comfort levels but resulted in higher treatment costs. This addition of flexible biocompatible resin improved the mechanical properties, while the addition of cranberry extract improved the biological properties.

While phytotherapy can be a promising approach, it is important to note that evidence supporting the use of these natural remedies for DS is still evolving. Further research is required to establish their efficacy and safety. Additionally, the severity of the condition and individual patient factors should be considered when determining the most appropriate treatment approach for DS [24].

Notably, the study involved a limited number of subjects and a short-term evaluation through confocal microscopy on the denture bases and salivary candidal CFU assessment. Consequently, long-term follow-up studies involving a larger sample size are imperative for future research endeavors.

## Conclusions

The following conclusions can be inferred on the basis of investigation constraints: (1) The study observed a statistically significant decrease in CFU at a concentration of 104 for 3D-printed dentures compared to PMMA dentures; (2) biofilm thickness was significantly lower in cranberry-infused 3D-printed dentures. The biofilm thickness and surface coverage showed statistically significant differences between PMMA and 3D-printed dentures. However, the volume fraction of biofilm did not exhibit a significant variation between the two groups; (3) the cranberry-infused 3D-printed dentures demonstrated higher flexural strength than traditional PMMA dentures. Fracture toughness also showed a statistically significant improvement in 3D-printed dentures.

The findings suggest that incorporating cranberry extract into 3D-printable resins can be an effective strategy for reducing the risk of DS. Further study and long-term clinical analysis are recommended to verify the durability and clinical efficacy of cranberry-infused 3D-printed dentures. This study highlights the potential of cranberry-infused 3D-printed dentures in addressing denture-related challenges, offering improved microbiological and mechanical properties. The integration of advanced materials and technologies in dentistry opens new avenues for enhancing patient outcomes and oral health.
